# Wearable Sensor Node for Safety Improvement in Workplaces: Technology Assessment in a Simulated Environment

**DOI:** 10.3390/s24154993

**Published:** 2024-08-01

**Authors:** Fabrizio Formisano, Michele Dellutri, Ettore Massera, Antonio Del Giudice, Luigi Barretta, Girolamo Di Francia

**Affiliations:** 1 ENEA, 80055 Portici, Italy; ettore.massera@enea.it (E.M.); antonio.delgiudice@enea.it (A.D.G.); girolamo.difrancia@enea.it (G.D.F.); 2 STMicroelectronics, 95121 Catania, Italy; michele.dellutri@st.com; 3 STMicroelectronics, 80022 Arzano, Italy; luigi.barretta@st.com

**Keywords:** wearable, smart personal protective equipment (PPE), safety, particulate matter (PM), gas exposure, IoT, sensor node, indoor outdoor localization

## Abstract

Personal protective equipment (PPE) has been universally recognized for its role in protecting workers from injuries and illnesses. Smart PPE integrates Internet of Things (IoT) technologies to enable continuous monitoring of workers and their surrounding environment, preventing undesirable events, facilitating rapid emergency response, and informing rescuers of potential hazards. This work presents a smart PPE system with a sensor node architecture designed to monitor workers and their surroundings. The sensor node is equipped with various sensors and communication capabilities, enabling the monitoring of specific gases (VOC, CO_2_, CO, O_2_), particulate matter (PM), temperature, humidity, positional information, audio signals, and body gestures. The system utilizes artificial intelligence algorithms to recognize patterns in worker activity that could lead to risky situations. Gas tests were conducted in a special chamber, positioning capabilities were tested indoors and outdoors, and the remaining sensors were tested in a simulated laboratory environment. This paper presents the sensor node architecture and the results of tests on target risky scenarios. The sensor node performed well in all situations, correctly signaling all cases that could lead to risky situations.

## 1. Introduction

According to the INAIL (Italian National Institute for Occupational Accident Insurance) annual report in 2022 [1], the number of reports of accidents at work submitted to the Institute in the year 2022 was 703,432, representing an increase of 139 thousand cases compared with 2021. There were 1208 reports of fatal accidents, a decrease of 15.2% compared with 2021. The reports of pathologies of professional origin rose by 18,164 (+25.1%). The occurrence of accidents in the workplace by mode is shown in Table 1; the most accidents were the result of falling from heights.

Even one fatal accident at work is too much; this is why research and improvement in this field are important and desirable. With the recent improvements in the reliability of cyber–physical systems, new applications can be conceived to make workplaces safer than they were before. WAs workers can be continuously monitored in workplaces utilizing modern intelligent systems, it is possible to prevent and warn workers of risks. Smart PPE devices are cyber–physical systems that incorporate communication, elaboration, and sensing components that are deeply intertwined to pursue a specific task. This work presents a smart PPE which, together with a communication infrastructure and a remote server for visualization and alarm, contributes to the mitigation of risks related to the duties of workers and eventually improves the overall working conditions in workplaces. The smart PPE device oversees some quantities in the environment in which the worker is operating to recognize and prevent uncontrollable or hazardous situations. There are studies in the literature that cover the topic of the design of smart PPE [2,3,4]. In [2], the authors proposed a system that measures some parameters such as humidity, temperature, acceleration, and gas and exploits artificial intelligence to optimize choices in terms of safety at work. Here, sensors were incorporated in the helmet and the belt of the worker. However, the system designed in [2] does not address the problem of locating the worker in the working environment. The smart PPE presented in this work introduces an RTLS (real-time locating system) for indoor locations and a GPS for outdoor locations that lay the groundwork for an augmented reality remote assistant project covered in future works. In [3], a specific application is covered, namely the mitigation of risks in the case of workers who operate in construction sites where the main threat is represented by height. In [4], the authors presented a device that can monitor the concentration of carbon dioxide present in the air together with physiological sensors applied on the body of the worker; all this information is used to assess the worker’s state and, in case of anomaly, issue an alarm. The novelty in the present work, compared to the state of the art, is that the proposed solution offers an extensive sensor array and includes locating capabilities for both indoor and outdoor environments in a compact form factor. The node is equipped with a GPS sensor and an RTLS, the combined data of which are fed back to the control center with precise information about the position of the worker. Starting from that knowledge, in case of emergency, the rescuers can be informed of the exact position of the worker. The sensor node puts together a large mix of features, retaining reduced dimensions and low consumption. The features, among the others presented in the continuation of this article, include gas monitoring (VOC, CO_2_, CO, O_2_), air quality monitoring by means of a PM (particulate matter) sensor, a temperature and humidity sensor; in addition, the proposed device is equipped with a gyroscope, accelerometer, RTLS, GPS, LORA communication, and BLE. This work improves the preceding versions of the sensor node [5,6]. Some important improvements were made to achieve a compact form factor and improve portability. Low-power policies were introduced to improve battery endurance. A new localization system was implemented using two different localizing technologies, GPS and RTLS. Finally, a new experimental sensor array was integrated into the new node. Section 2 describes the details of the architecture, the sensors, and the devices used. Then, in Section 3, the results are shown along with the position testing. Section 4 is dedicated to the discussion of the results. Finally, conclusions and future work are reported in Section 5.

## 2. Materials and Methods

**Figure 1:**
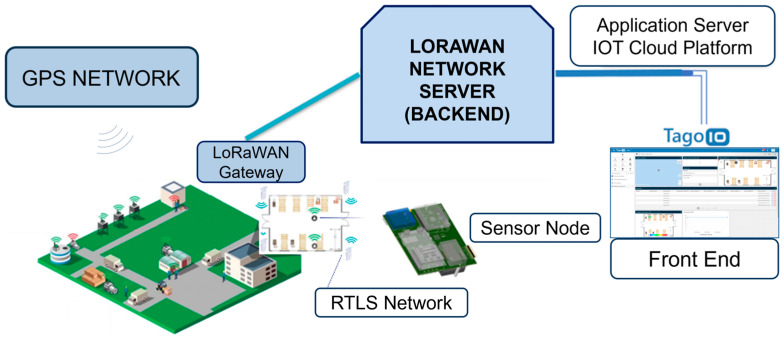
General architecture of the system from smart PPE to control room.

A sensor node was developed within an IoT architecture, enabling remote hazard detection in both indoor and outdoor environments. This architecture utilizes a real-time locating system (RTLS) combining ultra-wideband (UWB) and global positioning system (GPS) technologies (Figure 1). The figure depicts the entire system, including the sensor node (described in the next paragraph), an LoRaWAN™-based wireless network, and a remote-control center. The control center leverages the TagoIO cloud IoT platform (version 2.18.0) for data transfer, storage, and visualization. TagoIO was chosen due to its comprehensive suite of IoT data management features, including real-time alerts, data visualization, storage capabilities, and seamless integration with other platforms.

This architecture allows for remote worker status monitoring via a custom-designed visual interface within the TagoIO platform.

### 2.1. Signal Processing and Visualization

The node continuously monitors various sensor parameters, ensuring their compliance with predefined limits. Upon anomaly detection, the node transmits alarm or telemetry messages along with the worker’s location via an LoRa network.

The communication protocol leverages the standardized Cayenne Low-Power Payload (CayenneLLP) coding scheme. This approach guarantees compliance with payload size restrictions and enables the node to transmit diverse sensor data within a single message. Each data point is preceded by two bytes: a data channel identifier and a data type identifier. Table 2 details the payload structure.

During normal operation, the packet size is minimized to optimize transmission efficiency. Alarm status is represented by a 16-bit register, where each bit flag corresponds to a specific alarm type (refer to Table 3). This register is then embedded within a CayenneLLP packet that also includes worker location information and, in telemetry mode, the actual sensor value that triggers the alarm. This minimized payload size facilitates faster transmission, reduces on-network packet collisions, and allows for future expansion of the message format to accommodate additional data.

**Figure 2:**
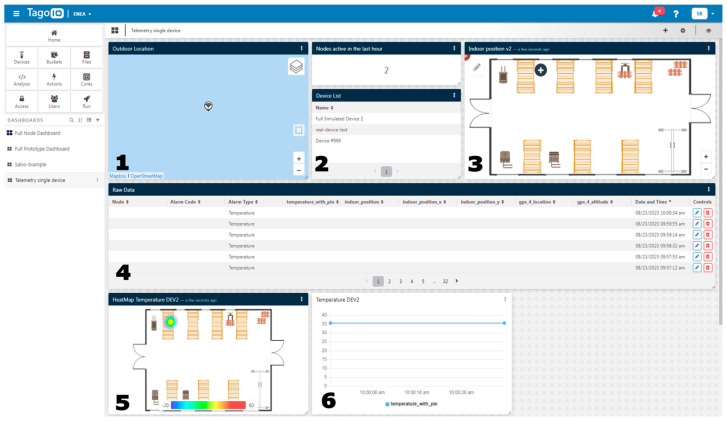
This picture shows the telemetry dashboard with the following info: 1. outdoor position, 2. active nodes, 3. indoor position, 4. raw sensor data, alarm type, and timestamp, 5. heat map, 6. data plot.

The received raw buffer, representing the encoded message, requires decoding to reach a human-readable format. This is achieved by employing a dedicated “Payload Formatter” software module (version 1.1), designed to decode newly received messages. The system dashboard (Figure 2) displays both alarm notifications generated by the node and a clear map depicting the worker’s location within the monitored environment. In the telemetry mode, the dashboard additionally displays data from various sensors alongside the standard information (alert, worker position, and timestamp).

### 2.2. Sensor Node Description

The IoT node’s core architecture comprises a multi-sensor node (MSN) and a communication board (CB). The MSN integrates sensors for environmental and motion monitoring, including temperature–humidity (HTS221), pressure (LPS22DF), and 6-axis inertial (LSM6DSOX) measurement. The CB incorporates the Teseo-LIV3F GNSS module for outdoor positioning and the STeval-STRKT01 LoRa connectivity module. To enhance air quality monitoring, PM (Plantower PMS 7003), VOC, CO_2_, CO, and O_2_ sensors are integrated. Indoor positioning is achieved through an external board in conjunction with fixed anchors. A dedicated audio board (STLCS01V1) processes noise levels, triggering alarms when they exceed safety thresholds. Instantaneous alerts are provided via buzzer, LED, and vibration motor.

**Figure 3:**
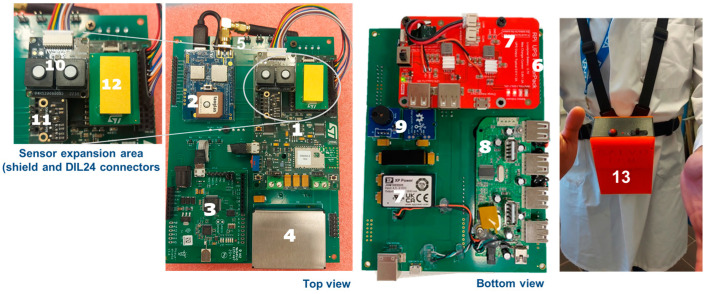
This picture shows hardware characteristics (block scheme and node photo); (1) MSN—multi sensor sub-node (STM customized board with sensor expansion area, shield, and DIL24 connectors); (2) CB—communication board, gps, and LoraWan tracker (STM STEVAL-STRKT01); (3) RTLS tag (POZYX tag); (4) PM—particulate matter sensor (PlantowerPMS7003); (5) PL, SL, MPS: power/status LEDs and main power switch connectors); (6) AB—smart audio board (STM STEVAL-STLCS01V1); (7) PMS—power management system (management and distribution); (8) HU—hub unit (power and data distribution unit); (9) BZ—buzzer; VM—vibration motor; (10) O_2_ and CO electrochemical solid polymer sensors (EC sense ES1-O2-25%, EC sense ES1-CO-1000 ppm); (11) VOC MOX sensor (Sensirion SGP40, 0–1000 ppm eth. Eq.); (12) experimental NDIR CO_2_ sensors; (13) final wearable sensor node. The PCB serves the primary function of interconnecting all the aforementioned boards and integrating the power management system (PMS). Individual power switches facilitate power-saving algorithms by enabling control over the power supply to each board. This allows for selective activation, such as engaging the real-time locating system (RTLS) solely during alarm situations. A lithium-ion battery with a capacity of 8000 mAh furnishes power to the system.

Prioritizing a compact, wearable form factor, a custom two-layer PCB minimizes external cables. The bare sensor node measures approximately 12 cm × 13 cm × 5 cm. Figure 3 illustrates the hardware architecture and the final packaged personal protective equipment (PPE), measuring 20 cm × 16 cm × 10 cm and weighing approximately 800 g with the 8000 mAh battery.

## 3. Results

The sensor node adopts a modular design, integrating dedicated sections for gas sensing, power management, localization, particulate matter detection, audio capture, and inertial measurement onto a single wiring motherboard. The node acquires data at predetermined sampling rates for various parameters and transmits it to a central control unit for visualization and data analysis.

To facilitate testing, the node’s functionalities are divided between the communication board (CB) and the measurement sensor node (MSN). This architecture enables independent testing of communication and sensor components but necessitates a synchronized communication protocol. An asynchronous leader–follower protocol is implemented, with the CB as the leader, initiating data exchange with the MSN.

Power consumption optimization is crucial due to varying sensor demands. The RTLS and communication modules consume the most power. To extend battery life, a hardware switch deactivates the RTLS when inactive, and the communication module enters a low-power state.

Initial testing revealed noise interference from the audio sensor, which was addressed by strategically positioning sensors to minimize disturbances. The audio sensor is placed away from noise sources, gas sensors are positioned for optimal airflow, and the temperature/humidity sensor is located in a cooler area.

### 3.1. Gas Detection Capability

The node incorporates commercial sensors. For CO and O_2_ sensing, EC sensors’ solid-state electrochemical sensors were selected based on TLV-STEL limits of 100 ppm for CO and 16% *v*/*v* for O_2_ [7]. The ES1-CO-1000 ppm and ES1-O2-25% models offer compact dimensions and low power consumption. For VOC sensing, the Sensirion MOX SGP40 model, with a range of 0–1000 ppm [8], was chosen. To meet the CO_2_ TLV-STEL of 30,000 ppm [9], a suitable sensor was selected. The gas sensor array is shown in Figure 3.

**Figure 4:**
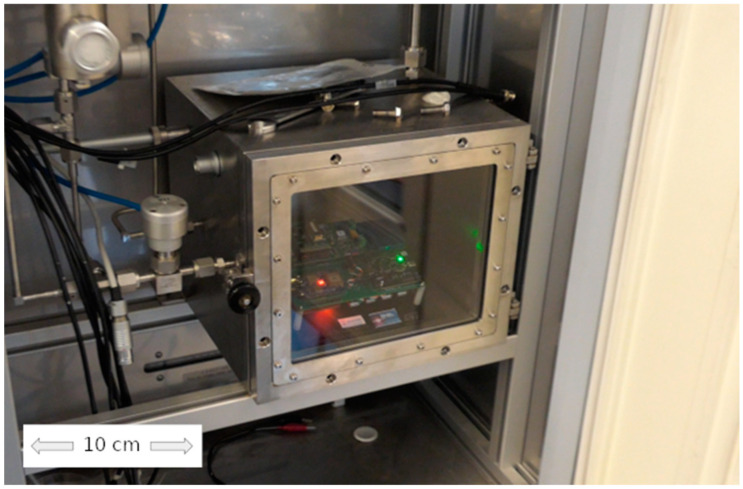
Sensor node installed in the gas sensing test chamber inside the GSCS equipment.

To evaluate sensor performance, extensive laboratory tests were conducted using a gas sensing characterization system (GSCS). This system simulates hazardous environments by controlling gas concentrations within a test chamber. The chamber, equipped with temperature, humidity, and pressure sensors, allows for precise atmosphere control. An artificial atmosphere is generated by mixing dry and wet air with the target gas, controlled by software (version 1.1) and C++ libraries. The sensor node was installed in the test chamber (Figure 4) and exposed to a constant airflow of controlled atmosphere at 22 ± 2 °C and 500 sccm.

**Figure 5:**
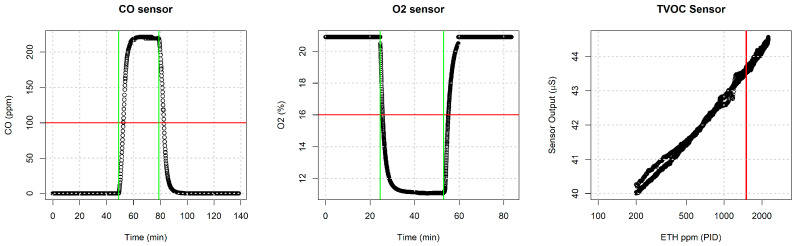
Sensor node gas sensors test in controlled environment: on the (**left**), see the time evolution of the CO-calibrated sensor response under an exposure of a concentration of 250 ppm of CO in synthetic air humified at 50% RH. The vertical green lines indicate the start injection and the stop injection while the horizontal red line indicates the alarm threshold; similarly, in the (**center**), see the O_2_ calibrated sensor response to an oxygen decrease in the artificial atmosphere; on the (**right**), see the ethanol sensor sensitivity curve from 200 ppm to 2000 ppm correlated to a calibrated PID. The red vertical line indicates the alarm threshold.

To assess gas sensor performance and verify alarm threshold accuracy, three tests were conducted, as illustrated in Figure 5. The tests involved sequentially exposing the sensor node to CO concentrations up to 250 ppm, reducing oxygen levels to 10%, and exposing it to ethanol concentrations exceeding 2000 ppm as a VOC representative. The red lines in the graphs indicate the sensor node’s alarm thresholds. Custom firmware was developed to extract data from each gas sensor for analysis.

The sensor node uses a prototype of an experimental NDIR CO_2_ sensor. The NDIR gas sensors have traditionally been larger in size compared to some other types of gas sensors, due to the need of longer optical path length and to place the source and the detector face to face, increasing the sensitivity. The gas sensor described in this work followed a different approach. It has been designed to fit into a DIL24 (dual in line) adapter board in order to be integrated into the IoT node.

The developed solution includes a flat lower internal wall, made of PCB (printed circuit board), to which the detector and the emitter are fixed, and a biconical upper internal wall which is configured to reflect the IR radiation issued by the emitter. Both the emitter and the detector face the upper internal wall so that the IR radiation is reflected by the upper internal wall before reaching the detector. Thanks to this reflection, the optical path is no longer formed by a single straight stretch, as mentioned before, and its total length is greater than the mutual distance between the emitter and the detector. This allows the size and bulk of the container body to be reduced while maintaining high absorption of IR radiation.

Thanks to the 3D simulation, the shape and size of the two reflectors have been optimized to achieve maximum sensitivity without increasing the overall dimensions. The shape of the lid is a biconical surface and coupling this surface occurs with a flat reflector on top of a PCB, which permits multiple reflections. Regardless of the direction of the emissions, every ray launched from the source hits at the detector and then is absorbed. 

Since the IR sensor itself is not capable of providing a radiation measurement that is frequency-selective, the first IR sensor also includes the optical filter, which allows the frequency of the IR radiation entering the sensor to be selected. In the case of CO_2_ detection, where the absorption wavelength of the main absorption peak is approximately 4.3 μm, the optical filter used is a bandpass filter from 4.2 μm to 4.4 μm. 

The second (reference) IR sensor differs from the first regarding the absorption range of the optical filter; in particular, at the reference wavelength, the absorption peaks of one or more gases to be detected are absent. For example, the reference wavelength can be equal to 3.89 μm; therefore, the absorption range of the optical filter of the second IR sensor can be between 3.79 μm and 3.99 μm approximately.

Reflecting the surface of the lid is achieved by standard gold plating, while the reflector on the PCB is achieved by rectangular metalization with NiAu plating.

**Figure 6:**
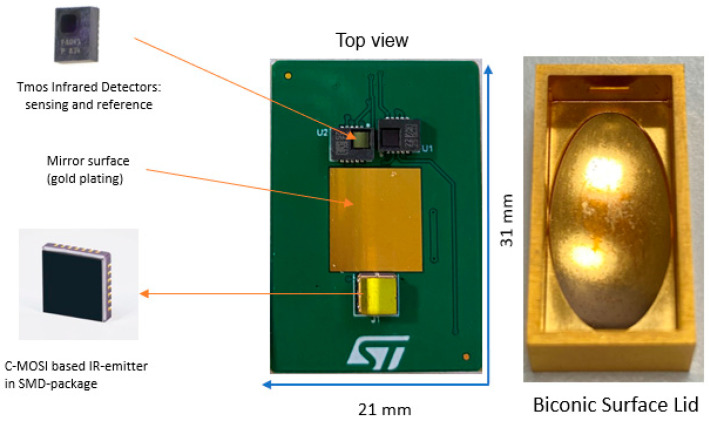
(**Left**): DIL24 PCB with reflecting surface; (**right**): internal shape of the lidded 3D rendering of the final wearable sensor node.

Figure 6 shows the DIL24 with a reflective surface and the internal shape of the lid.

To evaluate the performance of this sensor and thus understand whether it can meet the requirements of the scenario in which the multi-sensor node is to operate, we used the same instrumentation as for the characterization of the commercial gas sensors previously seen.

The node was exposed to a concentration of 10,000 ppm CO_2_ and its response in terms of resolution and noise was then evaluated.

**Figure 7:**
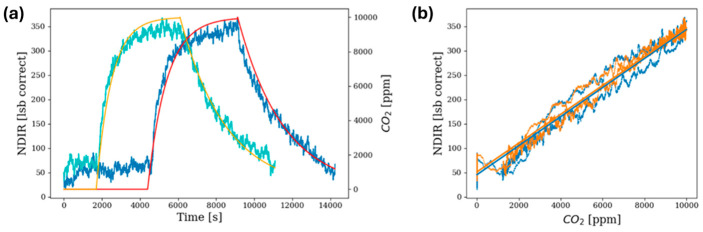
Calibration curves over time: (**a**) yellow and red show the concentration trends in the controlled chamber; green and blue show the relative sensor responses; (**b**) scatter plot between chamber concentration and sensor response in the two analyte injections considered, respectively, in orange and blue.

Figure 7a shows two different calibration curves to which we subjected the sensor. As can be seen, the sensor response time was determined to be significantly faster than the chamber’s estimated 1200 s response time, indicating rapid response to the target gas. This result allows us to state that the sensor responds to the analyte gas within a few seconds, and this is a first result that meets the specifications of the scenario.

On the other hand, as can be seen from the scatter plot in Figure 7b, the sensor exhibits a strong linear relationship with CO_2_ concentration within the tested range. The correlation coefficient between the values recorded in the chamber and the sensor response is close to 1. The sensitivity recorded is 30 ppm per appreciable lsb of the sensor. However, in terms of noise, we estimated the noise from the baseline; therefore, when the sensor is in a ‘resting’ state (not exposed to the analyte), it is around 30 lsb. 

The sensor’s response to common disturbances such as temperature and humidity was also evaluated. Considering the temperature variation, a drift was recorded, which was easily corrected with a firmware recalibration, while no drift was recorded when the sensor was exposed to a relative humidity variation between 20 and 70%.

Considering the data obtained, the sensor under examination is certainly capable of meeting the specifications dictated by the scenarios in which it will operate, even if noise rejection can be improved working on the hardware components.

### 3.2. Power Management

To improve battery life, this study investigates and implements tailored power management strategies that address the node’s diverse operational scenarios.

Beyond employing low-power sensor solutions (e.g., electrochemical sensors, MEMS with onboard complex gesture recognition), component activation policies were introduced to ensure on-demand operation, minimizing unnecessary power consumption. Testing revealed that localization devices, specifically TAG RTLS, significantly impacted overall power consumption. Consequently, a key strategy involved minimizing their active mode time to strictly necessary periods for localization tasks. Similarly, communication operations were optimized by limiting data transmission to sampled values and enabling programmable transmission frequency based on application requirements, further reducing energy consumption.

To determine the optimal balance between operational endurance and battery capacity, a series of empirical trials were conducted. These trials measured the operating time from full battery charge to complete discharge following some power management policies (e.g., RTLS module continuously active vs. active on-demand, as shown in Table 4) while capturing a single alarm event. The trials were performed under the same operational mode to isolate the impact of specific policies. The final battery selection (8000 mAh) aimed to ensure a monitoring session of approximately one workday, even in the worst-case scenario without any power-saving features enabled. Implementing these features demonstrably extends operational endurance, allowing for extended operation periods in mobility. Further energy savings can be achieved by leveraging the sleep and deep sleep modes of specific node modules. Due to the independent operating states of these modules (e.g., GPS tracking and LoRaWAN™ communication devices), utilizing their native low-power and ultra-low-power states can significantly reduce current draw. For instance, switching from active operation to a low-power state can decrease current consumption from 60 mA to 200 uA, representing a substantial 36% energy saving compared to operation without power-saving policies. This highlights the significant potential for further optimization through strategic use of these built-in functionalities.

### 3.3. Localization

Localization is achieved through a dual technology system combining RTLS UWB for indoor and GPS for outdoor positioning. This approach enhances localization precision, enabling sub-meter accuracy even in complex outdoor environments.

**Figure 8:**
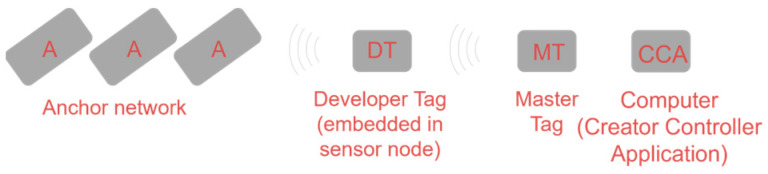
RTLS block scheme.

The RTLS UWB localization system is based on a commercial tag (POZYX developer tag [10]) incorporated in the node. The whole system, supporting the tag in the node, is a commercial product (Pozyx developer kit), which works through the dialogue of a master tag connected to a PC (both constituting the localization engine) and a constellation of anchors which are used as references for location measurements. Figure 8 illustrates the POZYX developer kit block diagram (source: POZYX product sheet).

**Figure 9:**
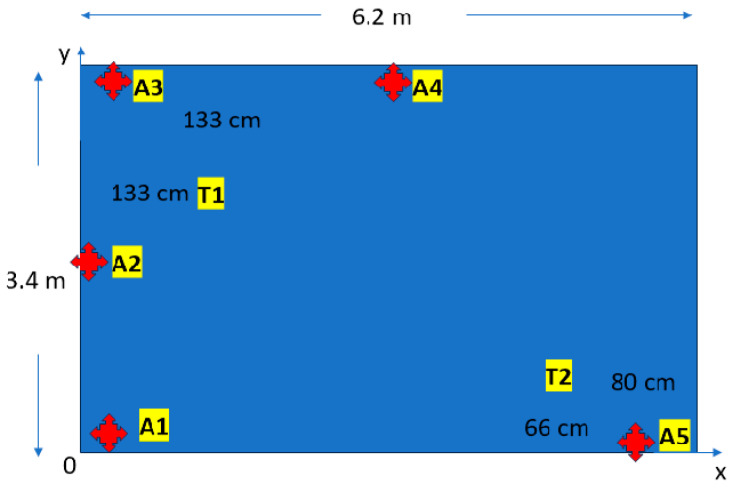
Two-dimensional map of the test room. In red, the anchors, T1 and T2 are the test points in the room.

In order to assess the precision and reliability of the localization system, a series of experiments were conducted within a controlled indoor environment. In the outdoor case the locating capabilities are entrusted to the performance of GPS system in a two-dimensional plane. The indoor case contemplates three-dimensional positioning. The test area (Figure 9) was a rectangular room measuring 6.2 m by 3.4 m, characterized by the presence of typical indoor obstacles such as chairs, cabinets, and various tools. The room is quite noisy in terms of electromagnetic radiation. To establish a reference frame for the localization data, a Cartesian coordinate system was defined within the room. The origin of this coordinate system was situated at the lower left corner of the room. The pavement corresponds to the *x*–*y* plane and the height corresponds to the *z*-axis. The *x*-axis coincides with the south wall and the *y*-axis coincides with the west wall. Five anchors have been placed in the locations shown in Figure 9. Two distinct test positions, labeled T1 and T2, were established within the room to evaluate the system’s performance under different conditions, while the anchors have been indicated with red cross and the capital letter A. The anchors have different heights from the pavement, A1 @ 1.5 m, A2 @ 1.6 m, A3 @ 1.5 m, A4 @ 1.9 m, and A5 @ 1.9 m. T1 (the node position for the test 1) is placed 133 cm from the north wall and 133 cm from the west wall and the sensor node has been elevated to 60 cm from the pavement. T2 is placed 66 cm from the south wall and 80 cm from the east wall and has been elevated to 180 cm. 

**Figure 10:**
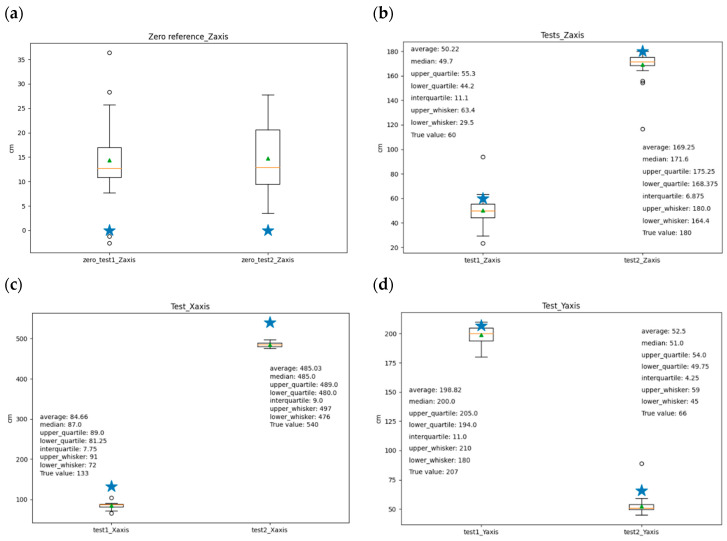
Boxplot representations of the test: the star is the true value, the triangle is the average value of the measures, and the line in the box is the median (2 quartile). Zero reference for the *z*-axis (**a**). Results for two tests on *z*, *x*, and *y*-axes, respectively (**b**–**d**).

Data analysis was performed using Python-based packages (v.3.6) including Seaborn, Matplotlib, NumPy, and Pandas. Initially, the zero-position offset was determined to remove systematic errors from subsequent position measurements. Analysis revealed accurate zero reference in the *x*–*y* plane, with errors within a 10 cm interval, but larger errors exceeding 10 cm in the *z*-axis (Figure 10a). Two node positions were then tested. In both cases, high precision was observed across all axes, as indicated by low variability and interquartile ranges in the box plots (Figure 10b–d). Considering the system’s application in worker localization and activity recognition, an error tolerance of 60 cm per axis was deemed acceptable for distinguishing between normal and abnormal working postures. Average measured positions were compared to true values (Table 5). While precision was satisfactory, accuracy was found to be limited, primarily due to the number and placement of anchors. The POZYX system manufacturer claims sub-10 cm accuracy under optimal conditions. To enhance accuracy, optimizing anchor positions is recommended.

### 3.4. Particulate Matter

To evaluate performance in high-particulate-matter conditions, the sensor node, equipped with a commercial Plantower PMS7003 dust sensor, was placed within a 500 L sealed chamber. A variable amount of certified dry Arizona Test Dust (ISO 12103-1) was injected into the chamber using a TOPAS SAG411 generator. A certified DustTrack 8533 reference instrument was concurrently installed to provide a benchmark measurement of particulate concentration.

**Figure 11:**
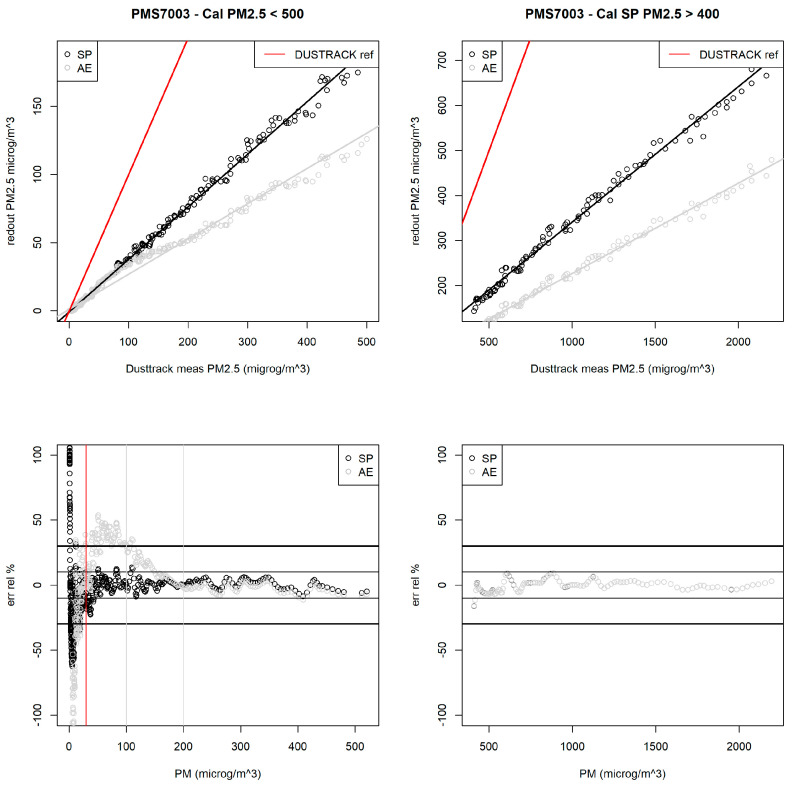
Sensor node PM measurement accuracy and precision: compared to reference instrument. The (**a**,**b**) images show how the Plantower PMS7003, installed on the sensor node, estimates the real value of PM2.5, measured by the reference instrument in two ranges: on the (**a**,**c**) from 0 μg/m^3^ to 500 μg/m^3^ and on the (**b**,**d**) from 500 μg/m^3^ to 2000 μg/m^3^. The two graphs at the (**c**,**d**) refer to the relative error that the PMS7003 made in a PM2.5 measurement after the recalibration. SP and AE refer to two different types of calibrated output.

The sensor node’s accuracy and precision were assessed across two PM2.5 concentration intervals: 0–500 µg/m^3^ and 500–2000 µg/m^3^. Figure 11 (top left and right) illustrates the linear correlation between measured and reference PM2.5 values. The sensor provides two calibrated outputs: SP and AE, reflecting different calibration standards. While the sensor demonstrates precision in high dust concentrations, accuracy diminishes, indicated by the increasing discrepancy between measured and reference values. This loss of sensitivity is likely attributed to the high concentration of submicron particles attenuating the laser beam used for measurement. Following recalibration, measurement error was reduced to an average of less than 30%, as shown in Figure 11 (bottom left and right).

### 3.5. Audio

**Figure 12:**
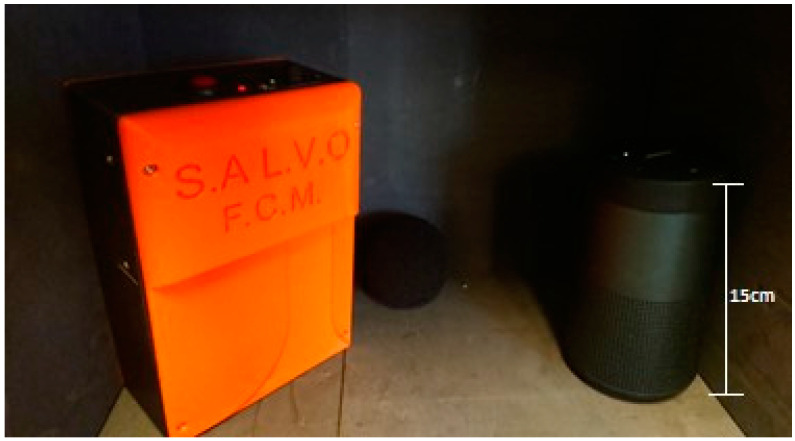
Audio test setup.

The sensor node incorporates an audio section for real-time environmental noise monitoring. A commercially available sensor tile board (STLCS01V1) equipped with a low-power, omnidirectional, digital MEMS microphone (MP34DT05-A) is employed for this purpose. The microphone’s capacitive sensing element, integrated circuit interface, low distortion, and high signal-to-noise ratio (64 dB) contribute to precise sound pressure detection and accurate signal representation. Notably, the guaranteed sensitivity of −26 dB ± 3 dB ensures a wide dynamic range for capturing diverse sound levels. The microphone’s reliable operation across a temperature range of −40 °C to +85 °C guarantees consistent performance in various environments. The audio section autonomously measures ambient sound pressure, triggering an alert when exceeding a predefined threshold. This threshold corresponds to noise levels mandating personal protective equipment (PPE) use. The sensor tile board enables not only real-time monitoring but also potential expansion to advanced audio analysis, adapting the sensor node to evolving requirements and enabling more sophisticated noise characterization. The audio section’s performance was evaluated in a dedicated test chamber (Figure 12). 

A test setup was established comprising the sensor node, a reference-grade Deltha Ohm HD2010UC phonometer, and a Bluetooth speaker for generating controlled sound stimuli. The speaker emitted sound frequencies ranging from 500 Hz to 4000 Hz in 500 Hz increments. Sound pressure levels were simultaneously monitored by both the phonometer and sensor node. The sensor node demonstrated exceptional performance, accurately detecting all sound events exceeding the 85 dB(A) threshold across the tested frequency range. These results confirm the sensor node’s reliable sound pressure detection capabilities.

### 3.6. Accelerometer and Gyroscope

The LSM6DSOX, a 3D digital accelerometer and gyroscope, was selected for its configurable full-scale acceleration (±2 g, ±4 g, ±8 g, ±16 g) and angular rate (±125 dps, ±250 dps, ±500 dps, ±1000 dps, ±2000 dps) ranges, high mechanical shock tolerance, and embedded complex gesture recognition capabilities. The sensor’s integrated pre-programmed free-fall detection feature, which leverages a dedicated register configuration to identify zero-gravity conditions, was of particular interest. To evaluate this feature, the sensor node was dropped from a height of 30 cm thirty times. Free-fall was accurately detected in all trials, triggering an appropriate alarm on the dashboard.

## 4. Discussion

This work introduces a novel personal protective device designed to enhance workplace safety and risk awareness. The device collects real-time data to enable data-driven security, monitoring worker actions and utilizing historical data with intelligent algorithms to prevent accidents. Given the safety-critical nature of the application, the hardware incorporates redundancies to ensure accurate environmental data.

Previous sections detailed the sensor node’s performance in individual components. Gas sensor tests, conducted in a controlled chamber, successfully detected CO levels exceeding the 100 ppm threshold. Similar tests using ethanol as a TVOC marker demonstrated strong correlation between sensor and reference instrument outputs, enabling TVOC threshold detection.

Localization, which is essential for worker tracking, was achieved through a combination of RTLS and GPS technologies. Indoor accuracy was influenced by anchor placement, while outdoor performance relied on GPS. A target localization accuracy of 5 m was established to support worker rescue and context-aware safety measures.

The sensor node’s particulate matter sensor accurately measured PM2.5 concentrations compared to a reference instrument, enabling the identification of excessive exposure levels. Early detection of elevated particulate matter allows for timely interventions to mitigate air pollution and protect worker health.

The integrated audio section monitored noise levels, triggering alerts when noise exceeded the predefined thresholds. This enables timely interventions to protect workers from excessive noise exposure. Additionally, the accelerometer and gyroscope detected falls and abnormal movements, initiating appropriate emergency responses.

Comprehensive system tests simulated real-world scenarios, including exposure to high noise levels and particulate matter concentrations. The system accurately detected these hazards, triggered alarms, and transmitted relevant data to the control center. Fall detection tests were also successful, demonstrating the system’s ability to identify and respond to emergency situations.

While the system demonstrated promising results, further optimization of anchor placement is necessary to improve indoor localization accuracy. Overall, the sensor node effectively collects and processes environmental data, enabling real-time monitoring and timely interventions to enhance worker safety.

### Security and Privacy Issues

The implementation of a system that monitors workers and their environment presents challenges beyond technical considerations. Paramount among these are the security of collected information and the protection of workers’ personal data. These factors are crucial in the widespread adoption of this technology in workplaces.

Smart PPE, integrating computing, communication, and artificial intelligence, offers dynamic hazard adaptation. To effectively manage emergencies, the system necessitates confidential, integral, and accessible data. Data confidentiality is essential to prevent unauthorized access and tampering, ensuring accurate decision making and intervention. Data integrity guarantees reliable and representative information for algorithm training and operational effectiveness. Data availability is crucial for timely responses to critical situations.

Privacy concerns arise from the device’s monitoring of worker position, movement, and audio. The collection of data ranging from device access logs to personal profiles raises questions about remote worker control. While legal exceptions exist for workplace safety and asset protection, navigating the evolving legal landscape and maintaining compliance can be complex and costly, particularly when relying on third-party software.

Comprehensive solutions addressing data security, privacy, and legal compliance are imperative for the widespread adoption of this technology. This paper focuses on technical aspects, recognizing the need for further research and development in these critical areas to fully realize the potential of smart PPE in enhancing workplace safety.

## 5. Conclusions

This work introduces a novel IoT sensor node for enhancing workplace safety. The system effectively collects and records environmental data, facilitating rapid intervention and worker alerts in hazardous situations. In emergency scenarios, it provides crucial information to rescue teams. Promising test results indicate its potential to significantly improve workplace safety when implemented and validated.

The sensor node incorporates a comprehensive suite of sensors, prioritizing wearability and extended battery life. Its dual-technology localization system enables precise indoor and outdoor positioning, while the multi-sensor array provides a holistic environmental assessment. These capabilities, combined with potential augmented reality integration, position the device as a pioneering solution within the emerging field of active PPE.

Future research will focus on device-to-device communication protocols, advanced AI algorithms for complex pattern recognition, and the development of a robust security and privacy framework to address legal and ethical considerations. By overcoming these challenges, this technology can be fully realized as a transformative tool for enhancing workplace safety.

## Figures and Tables

**Table 1:** Fatal accidents by mode of occurrence.

Height Fall	Object Fall	Loss Control Vehicles	Contact with Moving Objects	Vehicle Start	Contact with Washing Machines	Other
32.5%	16.8%	14.6%	6.8%	6.3%	5.5%	17.5%

**Table 2:** Payload coding.

1 Byte	1 Byte	N Byte	1 Byte	1 Byte	M Byte	…
Data 1 Ch.	Data 1 Type	Data 1	Data 2 Ch.	Data 2 Type	Data 2	**…**

**Table 3:** List of alarm coding.

Alarm Type	Register Value
Temperature alarm	xxxxxxxxxxxxxxx1
Humidity alarm	xxxxxxxxxxxxxx1x
Pressure alarm	xxxxxxxxxxxxx1xx
VOC alarm	xxxxxxxxxxxx1xxx
CO_2_	xxxxxxxxxxx1xxxx
PMS alarm	xxxxxxxxxx1xxxxx
Audio alarm	xxxxxxxxx1xxxxxx
BLE broadcast alarm	xxxxxxxx1xxxxxxx
Fall alarm	xxxxxxx1xxxxxxxx
User alarm	xxxxxx1xxxxxxxxx
-Unused-	xxxxx1xxxxxxxxxx
-Unused-	xxxx1xxxxxxxxxxx
-Unused-	xxx1xxxxxxxxxxxx
POZYX timeout	xx1xxxxxxxxxxxxx
POZYX sensor failures	x1xxxxxxxxxxxxxx
PMS sensor failures	1xxxxxxxxxxxxxxx
No alarms- No sensor failures	0000000000000000

**Table 4:** Battery comparison (RTLS module continuously active vs. active on-demand).

RTLS	Battery Capacity (mAh)	Endurance (h)	Battery Weight (g)
Continuously ON	8000	>7	121
Continuously ON	3800	<4	58
Continuously ON	6600	<6	97
ON-DEMAND	8000	>9	121
ON-DEMAND	3800	>5	58
ON-DEMAND	6600	>7	97

**Table 5:** Average of the measured samples versus true values.

	X	Y	Z
T1	84.66 vs. 133	198.82 vs. 207	50.22 vs. 60
T2	485.03 vs. 540	52.5 vs. 66	169.25 vs. 180

## Data Availability

No data available for disclosure.
